# The Book World of Medicine and Science

**Published:** 1903-02-28

**Authors:** 


### THE BOOK WORLD OF MEDICINE AND SCIENCE.

AIDS TO FORENSIC MEDICINE AND TOXICOLOGY. By WILLIAM MURRELL, M.D., F.R.C.P. Sixth Edition. (London: Baillière, Tindall and Cox. 1903. Price 2s. 6d. cloth, 2s. paper.)

No better description of the scope of this little book can be given than that which is contained in its preface. "This book will prove useful to students if used in the right way. It is not intended as a substitute for ordinary text-books on forensic medicine or for lectures. It is intended simply to recall facts which have been acquired and previously digested. It should be taken in small doses three or four days before going in for examination." Used in this way we have no doubt whatever about the utility of this small and handy but curiously complete work. On almost the first page we come across a passage which is so pertinent to a subject which has lately been much discussed that it will well bear quoting. Speaking of the Coroner's Court, Dr. Murrell says that if the deceased were not attended in his last illness by a qualified medical practitioner "the Coroner may summon before him to give evidence any legally qualified practitioner in actual practice living in or near the place where the death happened. The Coroner may order the medical practitioner so summoned to make a post-mortem examination and to analyse the contents of the stomach and intestines, and it makes no difference whether he has the requisite knowledge to undertake such work or not. He may not have made a post-mortem examination for many years, and may know nothing of chemical analysis and the detection of poisons, but that is of no importance, and the Coroner is strictly within his legal right. Most people will agree with a recent writer who points out that 'few general practitioners are fitted by their experience for performing necropsies,' and that 'it is a fearful thing to think that a man's life may depend on the report of a necropsy made by any qualified man on the register, who generally has no pathological experience, yet dare not refuse to make the examination.'"

The first part of this book deals with forensic medicine, the second with toxicology, and a careful perusal of its pages will soon show the student how thoroughly the work of condensation has been done and how neatly every necessary bit of information is put in its proper place. It is only when the student already knows his subject, however, that he will be able to appreciate all this. The book is in fact a note-book—a very good note-book—and its proper place lies in the recalling and reviving of knowledge which has already been learned, but is already dim and half forgotten, crowded out of mind perhaps by other topics. This it does admirably well.

A MANUAL OF PERSONAL HYGIENE BY AMERICAN AUTHORS. Edited by WALTER L. PYLE, A.M., M.D. Illustrated. (Philadelphia: Saunders and Co. 1901. 8vo. pp. 344.)

IT is obvious from the preface, and from the introduction into the text of what is termed in that preface "concise but adequate discussion of the anatomy of the parts under consideration" that this manual is devised for the general, non-medical, reader. To the ordinary layman, to the mother of a household, or the schoolmaster, it is impossible to recommend this as a good text-book. Of 335 pages of text, 132 are devoted to diseases and abnormalities of the eye and ear; and 70 pages to a "hygiene of the brain and nervous system," which is chiefly a popular sketch of the symptoms of neurasthenia. To introduce one page and a half on the management of glass eyes leaves us wondering why sections on the hygiene of trusses and cork legs have been omitted. The remarks on glass eyes would be excellent if they formed part of a student's manual on ophthalmic practice; but it must be a little mystical to the general reader to learn that free motility of a stump is "particularly the case where abscission, evisceration, implantation, or Mules's operation has been performed." The remarks devoted to the simple forms of ametropia and asthenopia are sufficient and well arranged, but there is a redundancy of unnecessary matter in these special subjects, and plates of the fundus occuli and pictures afforded by the laryngoscope and rhinoscope are not needed, and tend to a morbid introspection. We turned automatically to the description of the Lady Hypochondria in the Castle of Indolence:

> "She felt or fancied in her fluttering mood
> All the diseases which the 'spitals know,"

and we recalled the old controversy over the four last stanzas of the poem; that they were not written by Thomson, but by the poet physician whose chief claim to memory lies in his "Art of Preserving Health." Armstrong's treatise is in the laboured blank verse of 1744, and his teaching is simple, being solely the inculcation of temperance, soberness, and charity, coupled with plenty of exercise and good food. From every point of view we prefer the simple, ignorant, but scholarly verses of Armstrong to the over-specialised, ill-balanced material, offered in slipshod, jerky English, of the volume before us.

